# Open Retrograde Stenting of Proximal Innominate and Common Carotid Artery Stenosis

**DOI:** 10.3390/jpm14030223

**Published:** 2024-02-20

**Authors:** Marvin Kapalla, Albert Busch, Steffen Wolk, Christian Reeps

**Affiliations:** Department of Visceral, Thoracic and Vascular Surgery, University Hospital Carl Gustav Carus, Technical University of Dresden—TU Dresden, 01307 Dresden, Germany

**Keywords:** carotid endarterectomy, carotid stenting, hybrid carotid procedures, tandem carotid lesions, retrograde carotid stenting

## Abstract

Purpose: The evidence regarding the treatment of open retrograde stenting of innominate artery (IA) or common carotid artery stenosis (CCA) is limited, and is suspected to carry a high risk of stroke and death. Therefore, the objective of this study was to evaluate the outcomes of such hybrid procedures. Methods: A retrospective, monocentric study of all patients who underwent retrograde stenting of proximal IA and CCA stenosis via surgical cutdown of the CCA, with or without concomitant CEA, between 2016 and 2023 was performed. Results: Overall, 33 patients were treated. A total of 15 patients (45.5%) were male, with the mean age being 67 ± 9.1 years, and 58% (n = 19) of the patients presented with neurological symptoms. Open retrograde stenting was performed in 67% (n = 22) in ACC, and in 33% (n = 11) in IA stenosis. A total of 20 patients (61%) underwent retrograde stenting with synchronous ipsilateral CEA for concomitant stenosis of the carotid bifurcation. There was no 30-day mortality. The perioperative stroke rate was 3% (n = 1) with complete symptom recovery. During the follow up at 32 months (95% CI: 24–39), three late deaths (9.1%) and one symptomatic stent occlusion were observed and, in five patients (15.2%), re-intervention for restenosis was necessary. Conclusions: Open retrograde stenting for of proximal IA or CCA stenosis with or without CEA, in case of tandem carotid lesions, can be performed safely with a low rate of early adverse events. Continuous follow up examinations are necessary due to relevant instent re-stenosis rates.

## 1. Introduction

Stenosis, or occlusion, of the innominate artery (IA) and the proximal common carotid artery (CCA) is rare, with an crude incidence of 0.5–6.4% [1]. “Tandem-disease”, where the lesion affects both the internal carotid artery (ICA) and a proximal aortic arch branch (either the IA or CCA), has been reported with a prevalence of up to 4.3% in patients with cerebrovascular disease [2]. Thereby, stenosis, or occlusion, of the IA or the CCA may present with different clinical symptoms in the ipsilateral carotid territory, such as a right-sided subclavian steal syndrome or vertebrobasilar insufficiency [3].

The treatment options for such proximal aortic arch branch lesions range from open surgery that requires a sternotomy to the much less invasive percutaneous transfemoral, or transradial, endovascular procedures. These less invasive procedures come with a relevant risk of stroke in patients with high atherosclerotic burden, heavy calcifications, and/or complex lesions. In the cases of these patients, hybrid open retrograde stenting of the common carotid and innominate artery stenosis potentially offers better cerebral protection through avoiding passage through the aortic arch and, most important, through intermittent CCA clamping and flushing during and after the cannulation and stenting procedure, respectively. In particular, tandem carotid stenosis is, therefore, predestined for a hybrid approach through a single cervical access [4,5]. Unfortunately, a case series and a Vascular Quality Initiative^®^ (VQI) database review (66,519 procedures) described that the simultaneous implementation of a carotid endarterectomy and proximal ipsilateral retrograde CCA stenting for tandem carotid stenosis carries a high risk of stroke and mortality (combined stroke and mortality rate up to 11.3%) [6,7,8,9,10,11]. It is important to note that up to 20% of patients can show anatomical variations in the outlet of the supra-aortic vessels [12]. In a review, Popieluszko et al. were able to demonstrate that the bovine arch (a common trunk giving rise to the IA and the left ACC, 13.6%) is the most common variation. This variation of the common carotid variation (common trunk of the right and left ACC, 0.3%) can complicate the treatment procedure and, therefore, must be considered during planning due to the increased risk of cerebral embolization [12,13,14]. An aberrant right subclavian artery (arteria lusoria, prevalence around 1%) is a rare anatomical variant of the aortic arch which must also be taken into account during surgical procedures [15]. However, available data on the treatment in the normal anatomies of these pathologies in hybrid procedures, such as an open retrograde stent angioplasty, are still limited [4,7], and the basic endovascular expertise in the reporting centers is not known. Nevertheless, the current guidelines of the European Society for Vascular Surgery (ESVS) recommend considering an open retrograde stenting for symptomatic patients with proximal IA or CCA stenosis, but the treatment of asymptomatic patients, in particular, has limited evidence and remains controversial [16].

The aim of this study was, therefore, to evaluate the perioperative results of open retrograde transcarotid stent angioplasty in patients with or without tandem lesions of the ipsilateral ICA, as well as to assess the midterm-term results.

## 2. Methods

### 2.1. Data Collection and Study Population

All patients undergoing open retrograde stenting of proximal IA or CCA stenosis, with or without CEA, between November 2016 and November 2023, in the Division for Vascular and Endovascular surgery in the Department for Visceral, Thoracic, and Vascular Surgery at the Carl Gustav Carus University Hospital in Dresden, were retrospectively reviewed. The data for each case was retrospectively analyzed based on electronic patient records and imaging. Demographics, comorbidities, radiologic data, treatment modalities, complications, length of hospital stay, and follow up examinations were collected. During follow-up stent patency, re-interventions and neurological events were recorded. Exclusion criteria were patients receiving open retrograde stenting in the context of complex endovascular aortic procedures (e.g., arch endoprosthesis, chimney TEVAR), as well as isolated CEA, isolated subclavian artery stenosis, and proximal supra-aortic stenosis treated via transthoracic approach.

### 2.2. Ethics Approval

All procedures in the studies involving human participants complied with the ethical standards of the institutional research committee. Under the guidelines for research on human subjects, the local ethics committee at the Technische Universität Dresden approved the study (decision number BO-EK-427102023). The ethics committee is registered as institutional review board (IRB) at the Office for Human Research Protections (OHRP) (registration number IRB00001473 and IORG0001076). 

### 2.3. Diagnostics and Treatment Indications

All patients were preoperatively assessed by an interdisciplinary carotid-vascular board meeting that consisted of vascular surgeons, neuroradiologists, angiologists, neurologists, and interventional radiologists. Duplex sonography was primarily performed, and the degree of stenosis was evaluated by the peak systolic and end-diastolic velocity in all extracranial and extrathoracic vessels according to the criteria of the North American Symptomatic Carotid Endarterectomy Trial (NASCET) [16,17]. A validation of duplex sonography with a threshold PSV ≥ 250 cm/s and EDV ≥ 60 cm/s, to identify significant (>60%) CCA stenosis, was depicted by Matos et al., and was also used in our center as cut-off values for further imaging [18]. A verification of the diagnosis was performed via computed tomography angiography or magnetic resonance angiography, as recommended in the current ESVS guidelines [16].

Indication for surgical intervention was provided in symptomatic patients affecting the carotid (neurological symptom) and/or the subclavian vascular territory (critical limb ischemia, blood pressure difference > 20 mmHg with Subclavian-Steal-Syndrome) and with presence of ≥70% stenosis or occlusion of the IA or CCA. Stenoses were considered symptomatic if the symptoms had occurred within the six months prior to the boards’ decision. 

Asymptomatic patients with critical stenoses (≥70%) and a life expectancy of greater than five years were evaluated based on their individual risk profile in our interdisciplinary carotid-vascular board and, if applicable, were indicated for revascularization for stroke-risk reduction. Relevant risk factors included old silent infarcts (CT morphologic correlate), as well as soft plaques and progressive stenosis despite best medical treatment and serve supra-aortic multivessel disease.

In cases of tandem stenosis, the indications for concomitant ipsilateral CEA were an ipsilateral internal carotid artery stenosis of ≥70%, or ulcerated and/or unstable plaque morphology, according to the current European Society for Vascular Surgery (ESVS) guidelines [16]. 

As in randomized trials, a detailed neurological examination by a specialist for neurology was performed in the case of every single patient, pre- and postoperatively, according to the National Institutes of Health Stroke Scale (NIHSS) [19].

### 2.4. Procedure Technique and Postoperative Course

All procedures were performed in a hybrid operating room under general anesthesia. An open cervical approach along the sternocleidomastoid muscle at the level of the carotid bifurcation was performed. Patients were administered heparin in order to maintain activated clotting times (ACT) equal to or higher than 250 s (checked in 30-min intervals). Intraoperative neurologic monitoring via the measurement of brain oxygenation with near-infrared spectroscopy (NIRS) and somatosensory evoked potentials (SEPs) were applied. Indication for primary shunting was based on the surgeon’s preference in case of serve disease in the contralateral and/or vertebral system. Secondary shunting was performed in case of significant decrease in the NIRS and evoked potentials. If concomitant ipsilateral CEA was indicated, CEA was always performed before stenting and reconstructed by bovine pericardium patch plasty or eversion endarterectomy.

**Figure 1:**
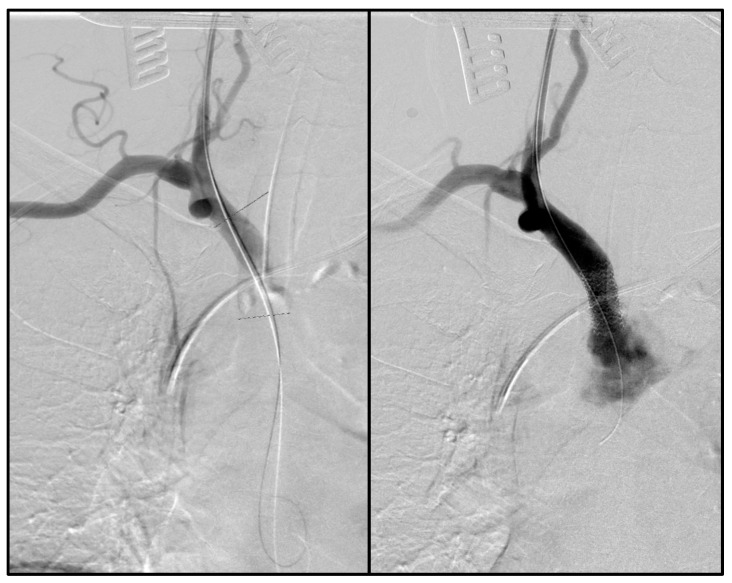
Intraoperative retrograde IA Stenting (**left**: before stent deployment, **right**: after stent deployment; dotted line: intraoperative marking for stent implantation).

For intervention, retrograde puncture of the CCA was performed followed by an introduction of a 7 French sheath (depending on the stent needed). In case of IA stenting, additional right-sided brachial access was implemented for stent deployment in case of kissing-stent procedure. Probing was performed routinely using a Terumo guidewire, which was replaced by a Supra Core™ (Abbott Laboratories, Chicago, IL, USA) guidewire, in complex lesions to achieve stable guidewire position for stent implantation. To avoid ostial wire crossing or extensive manipulation in the aortic arch, the wire was placed in the ascending aorta when probing the right CCA, and in the thoracic descending aorta when probing the left CCA. The wire was exchanged via a pigtail catheter or a Glidecath^®^ (Terumo, Somerset, NJ, USA) in the case of high-grade occlusions. A 7F sheath was then carefully advanced across the lesion to allow exact stent-graft placement. The stent was then implanted after retraction. Sheath placement, probing, and stent angioplasty were always performed under clamping of the ICA or distal CCA for cerebral embolic protection, and the vessel was flushed before cerebral blood-flow release. Balloon-expandable stents were routinely used due to their better deployment precision and greater radial force. The stent covering was decided based on the occlusion morphology. Covered stent-grafts were used for soft plaques with thrombotic coating, while uncovered stents were used for solid morphologies with severe calcification. As covered stent-grafts the Advanta V12 (Maquet-Atrium Medical Inc., Hudson, NH, USA), VBX (W.L. Gore & Associates, Flagstaff, AZ, USA), or iCover stent grafts (iVascular, Sant Vicenç dels Horts Barcelona) were selected based on surgeon’s choice and availability. As uncovered bare metal stents, the Express Vascular LD (Boston Scientific, Natick, MA, USA) was used. If necessary, post-dilatation was performed after stent implantation. During stent implantation, we did not reduce blood pressure and, instead, keep it elevated in order to achieve better cerebral perfusion via the opposite side and the cerebral arterial circle. An on-table completion angiography was routinely performed in all cases (Figure 1). A 12 French Redon drain was inserted, as is the standard, and this was removed on the second postoperative day depending on the amount of drainage. Postoperatively, all patients were admitted to an intensive care unit for at least 24 h for observation. 

Subsequently, patients received double antiplatelet therapy ASA 100 mg and clopidogrel 75 mg for at least 6 weeks without a loading dose. Thereafter, only aspirin was continued. In the case of a new postoperative neurological deficit, immediate imaging was performed routinely using computed tomography angiography (CTA) for exclusion of bleeding and vessel occlusion, followed by magnetic resonance tomography for identification of smaller cerebral lesions. Furthermore, a neurologist was involved regarding eventual stroke treatment. 

### 2.5. Outcome Parameters and Definitions

The primary outcome of this study was the combined perioperative stroke and mortality rate.

Secondary outcome parameters were technical success, overall-survival, patency, late neurologic events, and reintervention-rates during follow-up. Technical success was defined as a residual stenosis less than 20% of the target vessel on the final angiography. The perioperative period was defined as the first 30 days after treatment, or during hospital stay if the length was more than 30 days. Postoperative TIA was defined as a focal ischemic neurologic dysfunction with a duration of less than 24 h. Postoperative stroke was defined as any new ipsi- or contralateral neurologic event persisting over 24 h and correlating with new postoperative neuroradiological findings and increased NIHSS (NIHSS 1–4: minor stroke, NIHSS ≥ 5: major stroke) [19].

Complications were categorized according to the SVS reporting standards for carotid interventions and the Clavien-Dindo classification [20,21]. Primary and primary-assisted patency rates were calculated during follow-up. The follow-up period was from hospital discharge until the last available clinical examination. Routine follow-up consisted of clinical examination and duplex sonography every three and six months during the first year, and at least annually thereafter. If duplex sonography was not conclusive, or new relevant symptoms occurred, additional imaging (CTA) was indicated.

### 2.6. Statistical Analysis 

Statistical analysis was performed using IBM SPSS for Windows, Version 24.0 (IBM Corp., Armonk, NY, USA). All clinical characteristics were grouped so as to build categorical or nominal variables. Dichotomous variables were recorded as absolute frequencies (number of cases) and relative frequencies (percentages). Continuous data are presented as mean and standard deviation, non-symmetrical with median and interquartile range (IQR). Pearson’s chi-squared or Fisher’s exact test was used to analyze of categorical variables. Differences between means were tested with *t*-test or Mann-Whitney-U-test. Survival and patency data were analyzed using Kaplan-Meier estimates, and differences were appointed by the log-rank test. A two-sided *p*-value < 0.05 was considered statistically significant. 

## 3. Results

### 3.1. Study Population and Patient Characteristics

The study included 33 patients (54.5% male, age 67.12 ± 9.1 years; range 44–84 years). Overall, the patients presented with a high cardiovascular comorbidity, including arterial hypertension (n = 33, 100%), coronary heart disease (CHD) (n = 14, 42.4%), peripheral artery disease (PAOD) (n = 13, 39.4), and chronic kidney disease (CKD ≥ stage 4) (n = 7, 21.2%), as well as chronic obstructive pulmonary disease (COPD) (n = 13, 39.4%). Active nicotine abuse was documented in 51.5% (n = 17) of the patients, and five further patients (15.2%) had a history of smoking. One patient had previous (31 months) ipsilateral CEA. Further patients’ characteristics and comorbidities are summarized in Table 1.

Nineteen patients (57.6%) were symptomatic. Of these, four patients (12.1%) suffered from transient ischemic attack (TIA), eight (24.2%) from minor stroke (NIHSS 1–4), and three patients (9.1%) from major stroke (NIHSS ≥ 5). In addition, five patients showed symptoms in the subclavian artery territory (subclavian steal syndrome in n = 3 (9.1%) and acute arm ischemia (TASC I) in n = 2 (6.1%)). One patient showed both acute arm ischemia and a minor stroke (NIHSS 3). Duplex imaging and computed tomography angiography (CTA) were performed in all patients. Degrees of stenosis and vessel involvement are shown in Table 2. All treated lesions caused greater than 60% stenosis, and the majority of treated lesions caused greater than 70% stenosis (n= 27, 81.8%). The two patients (6.1%) with a 60% stenosis were treated due to apparent neurological symptoms. A vessel occlusion was seen in four patients (12.1%), each affecting the innominate artery. In total, the CCA was affected in 66.7% (n = 22) of the patients (right side 15.2% (n = 5) and left side 51.5% (n = 17)), and the IA in 11 patients (33.3%). A significant concomitant internal carotid artery stenosis (ICA) was detected in 20 patients (69.6%). Eight patients (24.2%) also had contra lateral ICA stenosis < 50%, without an indication for treatment.

### 3.2. Procedural Details

Overall, open retrograde stenting was performed in 67% (n = 22) of patients due to ACC, and in 33% (n = 11) due to IA stenosis. Twenty patients (60.6%) underwent retrograde stenting with ipsilateral CEA for tandem lesions (Table 3). Bovine pericardium patch plasty was used in 15 patients, and eversion endarterectomy in five. Intraoperative temporary shunting was implemented in four patients (12.1%), one due to neurological symptoms after clamping, and these were completely reversible after arterio-arterial temporary shunt establishment. Three primary shunt placements were performed in two cases due to severe preoperative neurological symptoms, and in one case due to serious heart failure (no possibility of intraoperative hypertension). Kissing stent implantation was conducted in 3 patients (9.1%) for severe IA stenosis. All stents used were balloon-expandable, this included covered stent grafts in 26 procedures (63.4%) and bare-metal stents in 15 (36.6%), with a stent diameter range from 7 to 18 mm. A total of 25 patients (75.8%) received one stent graft, and eight patients (24.2%) received two stent grafts. As covered stent-grafts Advanta V12 (Maquet-Atrium Medical Inc., Hudson, NH, USA) were implanted in 13 patients (39.4%), and VBX (W.L. Gore & Associates, Flagstaff, AZ, USA) in three patients (9.1%), as well as iCover (iVascular, Sant Vicenç dels Horts, Barcelona, Spain) in two patients (6.1%), and LifeStream (Becton Dickinson, Franklin Lakes, NJ, USA) in one patient (3%). Express Vascular LD (Boston Scientific, Natick, MA, USA), an as uncovered bare metal stent, was used in 14 patients (42.2%). Intraoperative technical success of target vessel treatment was 100%. 

### 3.3. Early Results (Perioperative)

In total, no in-hospital death or major stroke occurred perioperatively. There was only one perioperative ipsilateral minor stroke (NIHSS 4) with complete symptom regression during the subsequent inpatient stay, and another TIA suspicious postoperative event without lesions, or even cranial infarction, in immediate MRI diagnostics. Both events occurred in patients with a primary retrograde stent angioplasty of the IA without simultaneous CEA (direct open puncture of the CCA). Two symptomatic patients with simultaneous CEA suffered from cranial nerve injury (6.1%) as shown in Table 4. Both cranial nerve injuries were most likely incurred during ACC preparation. One asymptomatic patient developed an access site neck hematoma with a need for surgical evacuation. This was the only surgical complication in the treatment of asymptomatic patients. The mean hospital length of stay was 10 ± 6 days, and 1 ± 1 day in ICU. For asymptomatic patients, their length of stay was significant shorter (7 ± 4 days vs. 12 ± 7 days, *p* = 0.02). Within 30 days, there were no additional mortalities, new neurological events, or other complications recorded in the patient cohort.

### 3.4. Follow-Up

**Figure 2:**
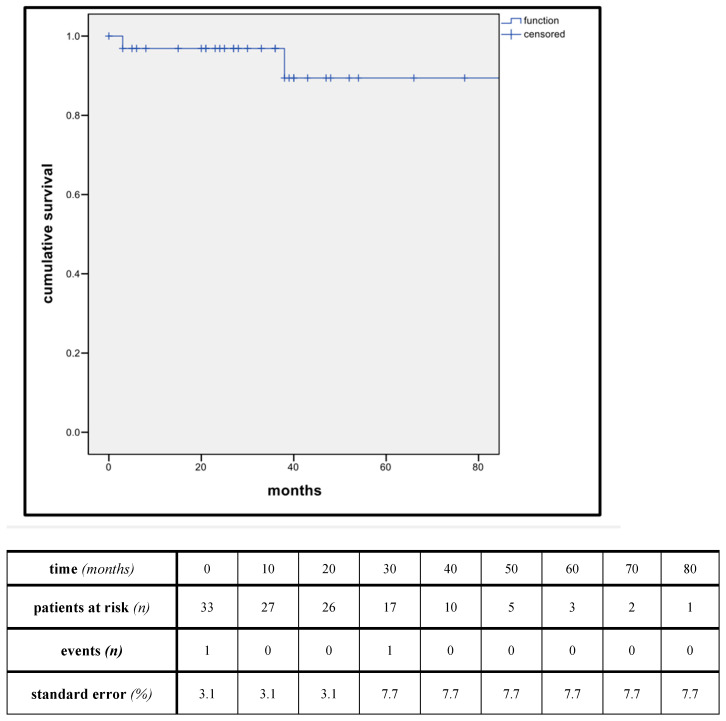
Kaplan-Meier estimates of overall survival. (estimator for median survival: 81.6 months, standard error (SE): 6.5%, 95% CI: 71–92 months).

**Figure 3:**
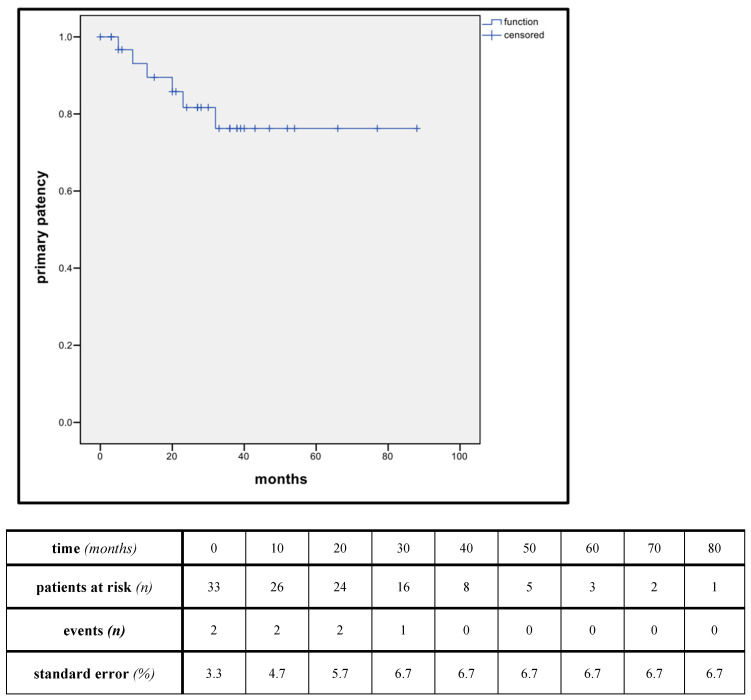
Kaplan-Meier estimates of primary patency. (estimator for median patency: 71.5 months, standard error (SE): 5.9%, 95% CI: 59–83 months).

The median follow-up was 32 months (range 0–88). Estimated Kaplan–Meier 1-year and 3-year survival manifested at 96.9% and 89.4%, while 1-year and 3-year primary patency manifested at 93.1% and 76.2% (Figure 2 and Figure 3).

In total, seven stent stenosis (21.2%), and one stent occlusion (3%), were observed during the follow-ups. Four stenoses and one occlusion (62.5%) occurred in symptomatic patients, and three re-stenosis (37.5%) were observed in asymptomatic treated patients. The one patient with left sided CCA stent occlusion after 22 months presented with fluctuating right-sided arm paresis, and underwent emergency surgery with carotid-subclavian bypass. They were subsequently asymptomatic. The stenosis in two were moderate (<60%), and there were five high-grade stent stenoses (≥70%). No significant difference was observed regarding the type of stent used, (n = 6 (75%) covered vs. n = 2 (25%) uncovered, *p* = 0.24). Overall, re-intervention was necessary in five patients (15.2%) due to significant re-stenosis (≥70%). Three of these five patients were initially treated with covered stents (60%), while two patients were initially treated with uncovered stents (40%) *p* = 0.79. With the exception of the symptomatic stent occlusion, all other four patients were asymptomatic regarding their re-stenosis. All five re-stenosis were successfully re-stented without new neurological deficit (three open retrograde and two transfemoral interventions). No other neurological event occurred during the follow-up. There were three late deaths (9.1%), which were not related to surgical treatment or a neurological event (COVID-19 pneumonia n = 1, cardiac n = 1, bronchial carcinoma n = 1). 

## 4. Discussion

This study demonstrates high technical success and safety with acceptable neurologic complication rates, as well as good mid- and long-term results in a large cohort of 33 patients who were treated with open retrograde stenting of the proximal supra-aortic branches, with or without concomitant carotid endarterectomy (CEA), for tandem carotid artery lesions [4,5,7,22,23].

In line with previous published meta-analyses from Robertson et al., and different single center experiences, our combined 30-day stroke and death rate of 3% was very low [3,4,7,24,25]. In contrast, two studies (single- and multicenter cohort) by Clouse et al. presented in higher rates for combined stroke and death of 9% and 11.3%, respectively [6,8]. Similar results were reported earlier by Sullivan et al., with 14% of periprocedural stroke rate [9]. However, all strokes in these series of cases occurred in patients with simultaneous ipsilateral CEA [6,8,9]. In comparison, our cohort notes a single minor stroke (3%) and one TIA (3%), and no perioperative death. Contrary to the published cases, both neurological events occurred after direct open retrograde stenting of the IA without concomitant CEA. In patients treated for tandem carotid artery lesions in our cohort, no complications were observed during early or late follow-up. In addition, rates of symptomatic patients were even higher in our cohort (58%) as compared to Clouse et al. (35%) [6]. 

Our single perioperative stroke occurred in a cardiovascular high-risk patient with supra-aortic multi-vessel-disease who was being treated for a symptomatic high-grade stenosis of the IA. Beach et al. assumed a higher perioperative risk for patients with contralateral occlusion based on data from the Society for Vascular Surgery registries, which reported an increased rate of adverse events (4.2% vs. 3.1%) and stroke (3.1% vs. 1.1%) in patients with contralateral occlusion [24,26]. However, this is not applicable to our cohort as all contralateral stenoses were not significant (<60%), and no contralateral occlusions were observed.

To prevent these perioperative neurological events, we always used distal cerebral protection by clamping the distal CCA or ICA, based on the location of the lesion. In the case of simultaneous CEA, we always performed the CEA first in order to allow distal CCA clamping and the reestablishment of the flow from the external to the internal carotid artery, as described similarly by several authors [24,27]. 

The largest meta-analysis to date, performed by Robertson et al. with 1.969 patients from 77 studies, compared hybrid open retrograde stenting (with simultaneous CEA) versus open surgery versus an isolated endovascular approach. The procedural risk was higher in the open surgical group (30-day death/stroke rate 7%) as compared to the hybrid (30-day death/stroke rate 3%) or endovascular group (30-day death/stroke rate 1.5%). Nevertheless, it was also shown that late re-stenosis was lowest for open surgery (2.6%) as compared to hybrid open retrograde stenting (10.5%) or isolated endovascular approach (9%) [7]. However, the authors themselves suspected a selection bias in the groups, given that the common presentation in the endovascular group were asymptomatic stenosis, and there was a remarkable lower proportion of IA stenosis as compared to the other groups. The increased perioperative morbidity in the open surgery group was also not surprising due to the invasiveness of the procedure and often more severe calcifications. Notably, the endovascular group also included 15.6% interventions with open CCA access (without) simultaneous CEA, and no tandem lesions were treated by an isolated endovascular approach rendering comparability even more difficult [7]. 

In the case of a tandem lesion, however, an increased periprocedural risk must be considered with an isolated endovascular approach, as a meta-analysis of four large RCTs (n = 6659) comparing CEA with CAS (carotid artery stenting) showed that the 30-day death/stroke rate was higher with 3.08% in CAS vs. 2.19% in CEA [10,19]. For these lesions, a hybrid approach should therefore be given priority [7].

As noted in other series, restenosis is common and is similar to the rate found in this study, with seven stent stenosis (21.2%) and one stent occlusion (3%) [8,24]. However, we experienced a low rate of neurological events in only one patient (3%) with a symptomatic left sided CCA stent occlusion after 22 months. The seven stent stenoses were asymptomatic, but five of them (15.2%) were high-grade (≥70%) and were reintervened without complications. All reinterventions took place within the first two years after primary treatment. This may support the importance of regular clinical and imaging controls during the follow-up, especially in the first year after the index procedure, as also recommended by Makaloski et al. [3]. Our approaches for reinterventions are high-grade restenosis (≥70%) confirmed by duplex sonography and CTA or symptomatology and, thus, with simultaneous indication as for the primary treatment. In contrast to Beach et al., we do not wait for symptom development and, instead, perform a prophylactic revascularization in cases of relevant re-stenosis to ideally reduce patient morbidity [24]. We observed a higher rate of re-stenosis in covered stent grafts (75% vs. 25%), but not on a significant level (*p* = 0.24). The increased re-stenosis rate is possibly attributable to the occlusion morphology, since we used covered stent grafts mainly for soft plaques with thrombotic coating, and these lesions were associated with an increased re-stenosis rate in some studies [28,29]. However, due to the size of the case series, we cannot draw any general conclusions about the comparison between covered and uncovered stents, and covered stents have shown improved long-term patency as compared to bare metal stents at different vascular beds in larger RCTs [30,31,32]. 

Especially in asymptomatic patients, the perioperative risk must be weighed against the risk of events from the lesions [24]. In our study, 42.2% were asymptomatic patients and they were treated without any complications or neurological events. In contrast, the current 2023 ESVS guidelines did not recommend treatment for asymptomatic patients (even with high grade stenosis), probably due to the low evidence and lack of data as a consensus recommendation [16]. However, they also recommend treatment for asymptomatic proximal ICA stenosis with CEA if the patients have a life expectancy ≥ 5 years and perioperative stroke/death rates of 3% or less [16]. Against this background, we find treatment of asymptomatic stenoses of the proximal CCA/IA under the same criteria appropriate in order to prevent neurological events which can be achieved safe with an open retrograde approach [3,4,7,24]. Finally, investigations on the natural course with best medical treatment of these CCA/IA lesions are needed in order to clarify this indication and evaluate the risk of neurological events in the cohort.

This study has some limitations. First, it is limited by the small number of cases and to the retrospective non-randomized single center study design, generating bias linked to a retrospective data collection, device, and patient selection. Furthermore, during the study period of seven years, there has been a learning progress and a gain in expertise regarding this endovascular technique that may have affected treatment procedures. Lastly, procedures might not be directly comparable due to confounding bias between operators. 

## 5. Conclusions

Open retrograde stenting of proximal IA or CCA stenosis with or without carotid endarterectomy can be performed with high technical success and low adverse events rates in a cardiovascular high-risk cohort. In particular, tandem lesions can be treated safely and without neurological events, especially in asymptomatic patients. Primary patency in long-term follow up appeared to be acceptable, but moderate re-intervention rates due to re-stenosis must be considered, and close follow-up is mandatory.

## Figures and Tables

**Table 1:** Demographic and clinical data.

Variable *	n = 33 (%)
**Demographic data**	
Age (years)	67.12 ± 9.1
Sex (male/female)	18/15 (54.5/45.5)
**Risk factors and comorbidities**	
Chronic kidney disease **	7 (21.2)
Heart failure (>NYHA II)	6 (18.2)
Atrial fibrillation	6 (18.2)
Hypertension	33 (100)
CHD	14 (42.4)
peripheral artery disease (≥Fontaine IIb)	13 (39.4)
COPD	13 (39.4)
Diabetes mellitus	14 (42.4)
Nicotine abuse (active)	17 (51.5)
Nicotine abuse (history)	5 (15.2)
Tumor disease	4 (12.1)
Previous carotid artery surgery	1 (3)
**Preoperative medication**	
Platelet inhibition	28 (84.8)
Oral anticoagulation	7 (21.2)
Statins	21 (63.6)

CHD, coronary heart disease; COPD, chronic obstructive pulmonary disease. * Continuous data presented as mean ± standard deviation; ** GFR < 30 mL/min/1.73 m^2^ (CKD stage ≥ stage 4); bold, subitems.

**Table 2:** Vessel’s involvement and preoperative symptoms.

Variables	n = 33 (%)
**Stenosis degree of target vessel**	
>60% and <70% stenosis	2 (6.1)
>70% stenosis	27 (81.8)
Occlusion	4 (12.1)
**Vessel location**	
Innominate artery (IA)	11 (33.3)
Right common carotid artery	5 (15.2)
Left common carotid artery	17 (51.5)
Concomitant significant internal carotid artery stenosis (ICA)	20 (60.6)
Concomitant contra lateral internal carotid artery stenosis (ICA)	8 (24.2)
**Preoperative symptomatic**	19 (57.6)
Transient ischemic attack (TIA)	4 (12.1)
Minor stroke (NIHSS 1–4)	8 (24.2)
Major stroke (NIHSS ≥ 5)	3 (9.1)
Subclavian steal syndrom	3 (9.1)
Acute arm ischemia (TASC I)	2 (6.1)
asymptomatic	14 (42.2)

NIHSS, National Institutes of Health Stroke Scale; TASC, Transatlantic Inter Society Consensus Working Group; bold, subitems.

**Table 3:** Procedural details.

Variables *	n = 33 (%)
**Approach**	
Cervical	33 (100)
Brachial (right)	3 (9.1)
**Simultaneous ipsilateral carotid endarterectomy**	**20** **(60.6)**
Reconstruction after carotid endarterectomy	
Bovine pericardial patch	15 (45.5)
Eversion endarterectomy	5 (15.5)
**Shunt**	**4** **(12.1)**
Primary	3 (9.1)
Secondary	1 (3)
**Endovascular procedures**	**33** **(100)**
Kissing stents	3 (9.1)
Balloon-expandable stents	41 (100)
Covered stents	26 (63.4)
Bare-metal stents	15 (36.6)
Number of stents: n = 1	25 (78.8)
Number of stents: n = 2	8 (24.2)
**Technical success**	**33** **(100)**
**Intraoperative details**	
Operating time, minuets, mean (SD)	170 ± 79
Contrast agent, mL, mean (SD)	76 ± 34
Fluoroscopy time, minuets, mean (SD)	6.1 ± 5.6

* Continuous data presented as mean ± standard deviation.; bold, subitems.

**Table 4:** Perioperative course.

Variables *	n = 33 (%)
**Procedure-related complications**	
Access site hematoma	1 (3)
Horner syndrome	1 (3)
Retropharyngeal hematoma	2 (6.1)
Vocal fold paresis	1 (3)
Cerebrovascular	2 (6.1)
Transient ischemic attack (TIA)	1 (3)
Minor stroke (NIHSS 1–4) with delayed recovery	1 (3)
**Systemic complications**	**-**
**Revisions**	**1 (3)**
Hematoma evacuation	1 (3)
**In-hospital data**	
in-hospital mortality	0 (0)
ICU stay, days, mean (SD)	1 ± 1
Total hospital stay, days, mean (SD)	10 ± 6

* Continuous data presented as mean ± standard deviation. NIHSS, National Institutes of Health Stroke Scale. bold, subitems.

## Data Availability

The data presented in this study are available on request from the corresponding author.
